# Effect of melatonin supplementation on endothelial function in heart failure with reduced ejection fraction: A randomized, double‐blinded clinical trial

**DOI:** 10.1002/clc.23682

**Published:** 2021-06-28

**Authors:** Shervin Ghaffari Hoseini, Kiyan Heshmat‐Ghahdarijani, Saeid Khosrawi, Mohammad Garakyaraghi, Davood Shafie, Hamidreza Roohafza, Marjan Mansourian, Elham Azizi, Yousof Gheisari, Masoumeh Sadeghi

**Affiliations:** ^1^ Isfahan Cardiovascular Research Center, Cardiovascular Research Institute Isfahan University of Medical Sciences Isfahan Iran; ^2^ Department of Physical Medicine and Rehabilitation, School of Medicine Isfahan University of Medical Sciences Isfahan Iran; ^3^ Heart Failure Research Center, Cardiovascular Research Institute Isfahan University of Medical Sciences Isfahan Iran; ^4^ Cardiac Rehabilitation Research Center, Cardiovascular Research Institute Isfahan University of Medical Sciences Isfahan Iran; ^5^ Regenerative Medicine Research Center Isfahan University of Medical Sciences Isfahan Iran

**Keywords:** blood pressure, endothelial dysfunction, heart failure with reduced ejection fraction, melatonin, oxidative stress

## Abstract

**Background:**

This study aimed to investigate the effect of melatonin supplementation on endothelial function in patients with heart failure with reduced ejection fraction (HFrEF).

**Methods:**

This is an analysis of the MeHR trial, a randomized double‐blinded placebo‐controlled clinical trial with two parallel arms of 1:1. Oral 10 mg melatonin tablets or placebo was administered for 24 weeks. Deference in the percentage of flow‐mediated dilatation (FMD) after the intervention was the primary outcome.

**Results:**

Ninety‐two patients were included in the study (age: 62.7±10.3 years, 87.0% male, ejection fraction (EF): 28.6±8.1). After adjustment for baseline FMD and age, a statistically significant difference in post‐treatment FMD in favor of the melatonin group was seen (estimated marginal means [95%CI], melatonin: 7.84% [6.69–8.98], placebo: 5.98% [4.84–7.12], *p* = .027). There was no significant difference in the mean of post‐treatment systolic/diastolic blood pressure, serum total antioxidant capacity, and serum malondialdehyde (MDA) between groups. Subgroup analysis showed significant improvement in FMD and MDA in the melatonin group in nondiabetic patients, while no difference was seen between study groups in diabetic patients.

**Conclusions:**

Melatonin supplementation in HFrEF might improve endothelial function; however, this beneficial effect might not be seen in diabetic patients.

## 1 INTRODUCTION

Endothelial dysfunction (ED), which is the imbalance between vasodilatation and vasoconstriction properties of the endothelium, has been accepted as an essential pathogenic mechanism in with reduced ejection fraction (HFrEF).^1^, ^2^ Indeed, ED can result from chronic hypoperfusion of the tissues and the imbalance between oxidative stress and antioxidant defense mechanisms in HFrEF.^3^ Nitric oxide (NO) is the main vasodilator molecule released by the endothelium and plays an important role in microvascular competence and tissue perfusion. Increased oxidative stress in heart failure (HF) is represented by increased formation of reactive oxygen species (ROS), which reduces the bioavailability of NO and negatively impacts the expression of endothelial NO synthase (eNOS).^3^

Flow‐mediated dilatation (FMD) is a common and acceptable method to assess endothelial function and a reliable predictor of worse clinical outcomes in patients with HFrEF.^4^ Other mechanisms such as angiotensin II pathway, sympathetic over activity, and chronic increase of inflammatory cytokines also contribute to the ED seen in HFrEF.^4^

Several studies have recently focused on pharmacological and non‐pharmacological interventions targeting ED in HF. Melatonin, the major secretion of the pineal gland, seems attractive for this purpose. In addition to its well‐known role of circadian and seasonal rhythm control, melatonin is recognized as a potent and multipurpose antioxidant, acting via both activation of antioxidant enzymes and scavenging ROS.^5^ Furthermore, melatonin has anti‐inflammatory properties,^6^ can antagonize angiotensin II pathways,^7^ and is shown in experimental studies to reduce catecholamine levels.^8^ Thus, melatonin might protect vascular endothelium or reverse ED by multiple mechanisms.^9^ Numerous clinical studies have examined this effect of melatonin in different illnesses. However, to the best of our knowledge, HF has not been the subject of these studies, and the effect of melatonin on the vascular endothelium is unknown in this syndrome.

Systolic and diastolic PB is also directly related to the endothelial functions of vasodilation and blood vessels relaxation;^10^ thus, BP is supposed to be affected by melatonin supplementation.

Therefore, this study aimed to assess melatonin's effects on endothelial function, serum oxidative stress markers, and BP in HFrEF.

## 2 MATERIALS AND METHODS

### 2.1 Trial design

We conducted a unicenter phase II prospective randomized double‐blinded placebo‐controlled clinical trial with two parallel arms of 1:1 allocation on patients with HFrEF (Melatonin in Heart failure with Reduced ejection fraction: MeHR trial) between January 2019 and August 2020. This trial's rationale and design are thoroughly described elsewhere,^11^ and the protocol has been registered in ClinicalTrials.gov (NCT03894683). The trial has been approved by the Ethical Committee of Isfahan University of Medical Sciences (IUMS) (IR.MUI.MED.REC.1397.067). Written informed consent was obtained from all patients. This study followed the declaration of Helsinki's ethical principles, and the report is prepared according to the Consolidated Standards of Reporting Trials (CONSORT) guideline.

### 2.2 Trial participants

Patients with HFrEF were recruited from outpatient clinics of Chamran cardiology hospital, the referral cardiology center in Isfahan, Iran. Participants' eligibility criteria are fully explained before.^11^ Briefly, stable patients over 18 years with a documented diagnosis of HFrEF and on optimum medication according to the 2016 European Society of Cardiology (ESC) guidelines were enrolled. No sex or HF type (ischemic or non‐ischemic) restriction was imposed. Those who had a history of hospitalization or a change in their drugs due to an acute exacerbation of HF or ischemic event during the past 3 months were excluded. Echocardiography and routine laboratory tests were performed for all patients before randomization to ensure EF < 40 and exclude end‐stage liver and renal diseases.

### 2.3 Randomization and blinding

An invited investigator who did not participate in the study generated the random sequence in block sizes of four by the website Randomization.com (http://www.randomization.com). She coded the melatonin and placebo boxes and placed the codes in opaque sealed envelopes. The placebo and melatonin tablets were the same in their shape, smell, and taste and were provided in similar boxes of 100 tablets. Thus, the investigators who enrolled the patients and randomized them to the groups, as well as all outcome assessors, were blinded to the groups of intervention.

### 2.4 Interventions

Patients in the melatonin group received 10 mg melatonin tablets ingested every night at bedtime for 24 weeks. The placebo group was administered with placebo tablets, which were ingested the same as melatonin. The melatonin and the placebo tablets were manufactured by Sepid Teb Pharmaceutical Company (Sepid Teb Co, Tehran, Iran). The purpose and the course of the study were thoroughly explained to the patients and they received one box of melatonin or placebo after randomization and baseline assessments. Patients were revisited at week 12 and received the second box of melatonin or placebo. Follow‐up calls were used to monitor patients' clinical status, drug related side effects, and adherence to the treatment, during the study.

Because of the coronavirus disease 2019 (COVID‐19) pandemic, we asked the last 16 patients not to attend the study center for the first follow‐up, and they received the second drug box by post at their home. We were in contact with them to assure drug delivery to them.

### 2.5 Outcomes

The primary outcome of this analysis of the MeHR trial was the change in FMD after 24 weeks of intervention. Other outcomes were change in serum oxidative stress markers, including malondialdehyde (MDA) and total antioxidant capacity (TAC) after the intervention, and systolic and diastolic BP changes at first and second follow‐up relative to the baseline.

FMD measurements were done according to the related guideline^12^ using high‐resolution B‐mode ultrasound (GE Vivid 3.0, General Electric Vingmed Ultrasound) and a high‐frequency vascular probe with continuous electrocardiogram monitoring. A single specialist who was blinded to the study groups conducted the echocardiography and FMD tests. All experiments were done between 8 and 11 a.m. after at least 8 h of fasting and avoiding physical exertion. FMD was calculated as the percentage of post‐ischemic change in the brachial artery diameter relative to its basement diameter.

To assess the intra‐observer variability, recorded images of randomly selected 30 brachial artery measurements were reanalyzed by the same cardiologist, and the intra‐class correlation coefficient of 0.95 was obtained for the baseline brachial artery diameter.

BP was measured before, at week 12 (first follow‐up), and at week 24 (second follow‐up) of the study. It was measured after the patient had at least 10 min of rest, at a sitting position in the right hand by a digital sphygmomanometer (Beurer, GmBH, Ulm, Germany) and a standard cuff, three times every 1 min. The average of the last two readings of the systolic and diastolic pressures was recorded.

Fasting venous blood samples (5 ml) were obtained from patients, with all blood samplings between 7:30 and 9 a.m. Samples were allowed to coagulate at room temperature for 10 min and then centrifuged at 3000 rpm, and the serum aliquots were stored at −80 until subsequent testing. The routine blood tests (FBS, lipid profile, and renal and liver function tests) were performed by a Hitachi 902 auto analyzer.

MDA and TAC were measured in the stored serum samples colorimetrically, following instructions of commercial kits (ZellBio GmbH, Ulm, Germany). Baseline N‐terminal pro b‐type natriuretic peptide (NT‐pro BNP) was quantified in stored serum samples by a commercial ELISA kit (Bioassay Technology Laboratory, Shanghai, China; E1239Hu) following the kit's instruction. Urinary 6‐sulfatoxymelatonin (MS) levels were determined in overnight urine samples; briefly, patients were instructed to discard their urine at 7 p.m. and collect every excreted urine in a sterile container up to 7 a.m. The urinary and serum MS was measure by an ELISA kit (Bioassay Technology Laboratory, E1045Hu, Shanghai, China). Overnight excreted MS was calculated by multiplying MS level and overnight urine output.

Clinical data, including the history of the present illness, cardiovascular diseases, comorbidities, drug history, and body weight and height, were documented at the baseline. The level of physical activity was determined by the International Physical Activity Questionnaire short form (IPAQ‐SF).

### 2.6 Sample size and statistical methods

The sample size needed to detect a difference of at least 2% in FMD between groups with a power of 80%, and a two‐sided significance of 5% was calculated based on a study from Oikonomou et al.^13^ with the standard deviation of 3%, which gave a sample size of at least 36 in each group.^14^

An intention to treat analysis approach was used for statistical analysis, and all patients with at least one baseline measurement were included in the analysis. Missing data completely met the assumptions for missing entirely at random. Data were analyzed using SPSS version 22 (IBM SPSS Statistics). The independent *t*‐test or Mann–Whitney U/Wilcoxon rank‐sum test was used to compare means between groups depending on the variables' distribution. Fisher's exact test or chi‐square was used to compare the frequency of categorical variables between groups. Analysis of covariance (ANCOVA) or generalized estimating equations (GEE) model was used for adjusted analysis. A *p*‐value of less than .05 with two‐sided tests was considered significant.

## 3 RESULTS

### 3.1 Study adherence and baseline characteristics

**FIGURE 1:**
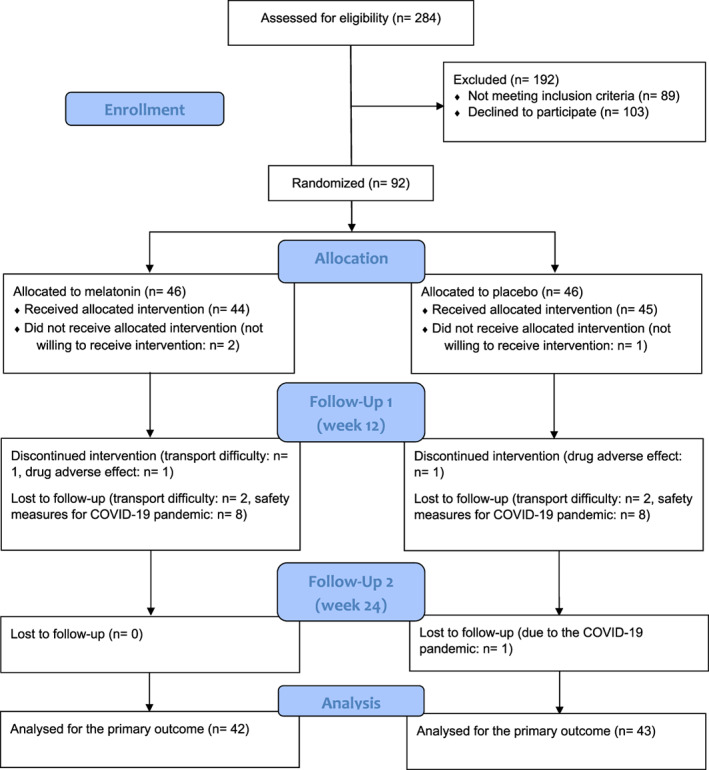
CONSORT flow diagram of the MeHR trial

A total of 284 patients with HFrEF were screened for eligibility criteria from January 2019 to February 2020, from those 92 patients (age: 30–82 years, male/female ratio: 80/12) were randomized to melatonin (n = 46) and placebo groups (n = 46) and finally, 85 patients completed the study and were followed for at least 24 weeks and up to 28 weeks. The flow diagram of the MeHR trial is presented in Figure 1.

The pill count at week 12 and 24 showed that administered drugs were appropriately consumed by the patients and only five patients (melatonin = 3, placebo = 2) discontinued the drug for more than five consecutive days (all less than 30 days) during the study.

Table 1 demonstrates the basic characteristics of the participants. Main clinical and demographic characteristics were balanced between two groups, except for diastolic BP and diuretic use.

**TABLE 1:** Socioeconomic and clinical characteristics of the participants

	Melatonin group (n = 46)	Placebo group (n = 46)	*p*‐value
Age (years)	62.7 (±10.3)	59.1 (±11.5)	.123
Sex (male)	40 (87.0%)	40 (87.0%)	1.000
Body mass index (kg/m^2^)	26.7 (±3.2)	27.2 (±4.3)	.541
Systolic BP (mmHg)	120 (106–135)	114 (105–131)	.131
Diastolic BP (mmHg)	77 (70–82)	74 (63–78)	.048
Medical history			
Time from diagnosis (months)	36 (12–84)	30 (13.8–60)	.943
HF etiology (ischemic)	42 (91.3%)	38 (82.6%)	.354
ICD/Pace	4 (8.7%)	9 (19.6%)	.231
Diabetes mellitus	16 (34.8%)	12 (26.1%)	.497
Renal disease	5 (10.9%)	1 (2.2%)	.203
Ever smoker	21 (45.7%)	24 (52.2%)	.677
Current smoker	9 (19.6%)	13 (28.3%)	.464
Opium dependence	7 (15.2%)	12 (26.1%)	.303
Medications			
ACEI/ARB	31 (67.4%)	32 (69.6%)	1.000
Beta‐blockers	34 (73.9%)	42 (91.3%)	.052
MRA	17 (37.0%)	20 (43.5%)	.671
Statins	38 (82.6%)	37 (80.4%)	1.000
Diuretics	15 (32.6%)	26 (56.5%)	.018
Digital	9 (19.6%)	5 (10.9%)	.385
Clinical and laboratory data			
LVEF (%)	28.46 (±8.3)	28.7 (±7.9)	.857
NYHA functional class			1.000
II	35 (76.1%)	35 (76.1%)	
III	11 (23.9%)	11 (23.9%)	
Physical activity level^a^			.654
Low	16 (34.8%)	12 (26.1%)	
Moderate	20 (43.5%)	22 (47.8%)	
Severe	10 (21.7%)	12 (26.1%)	
NT‐pro BNP (ng/L)	319 (280–374)	318 (281–375)	.985
FMD (%)	5.9 (2.2–9.2)	6.8 (3.2–9.8)	.317
Triglyceride (mg/dl)	132 (108–190)	126 (86–162)	.215
Cholesterol (mg/dl)	159 (138–190)	144 (132–181)	.303
HDL (mg/dl)	42 (36–50)	44 (40–49)	.380
LDL (mg/dl)	83 (70–104)	75 (63–99)	.263
TAC (μmol/L)	755.5 (592.8–855.5)	699.5 (578.8–799.3)	.144
MDA (μmol/L)	5.35 (3.97–7.02)	3.58 (2.37–5.70)	.011
Serum MS	123.9 (94.4–176.4)	131.4 (91.5–187.5)	.682
Overnight urine MS	6.4 (4.2–8.7)	7.0 (4.1–11.3)	.685

*Note:* Values are expressed as mean (±SD) or median (interquartile range) for continuous variables or number (percentage) for categorical data.

Abbreviations: ACEI, angiotensin‐converting enzyme inhibitors; ARB, angiotensin II receptor blockers; BP, blood pressure; FMD, flow‐mediated dilation; HDL, high‐density lipoproteins; HF, heart failure; ICD: implantable cardioverter defibrillator; LDL, low‐density lipoproteins; LVEF, left ventricular ejection fraction; MDA, malondialdehyde; MRA, mineralocorticoid receptor antagonists; MS, 6‐sulfatoxymelatonin; NT‐pro BNP, N‐terminal pro b‐type natriuretic peptide; NYHA: New York heart association classification; TAC, total antioxidant capacity.

a Determined by International Physical Activity Questionnaire short form (IPAQ‐SF).

### 3.2 FMD correlations

The correlation of the baseline FMD values with the potential covariates was examined. It was not correlated with the serum or overnight urine MS levels, the BMI, the systolic/diastolic BP, or the baseline NT‐Pro BNP values. Moreover, the baseline FMD values distribution was not statistically different according to the sex, etiology of HF, NYHA class, history of renal disease, smoking (past or current), opium use, and ACEI/ARB, beta‐blocker, MRA, or statin use. However, a negative correlation between the baseline FMD and the age (R = −0.217, *p* = .038) and a positive correlation with the left ventricular ejection fraction (LVEF) (R = 0.248, *p* = .017) was detected and the difference between the diabetic and nondiabetic groups approached to significant (*p* = .067).

### 3.3 Effect of melatonin supplementation on FMD

**FIGURE 2:**
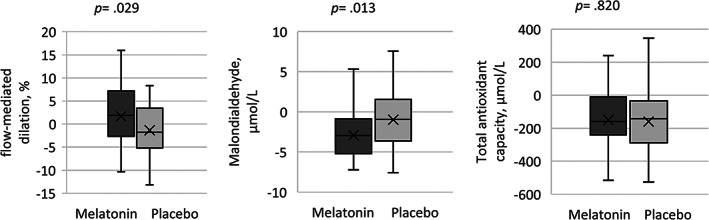
Effect of melatonin supplementation on FMD, MDA, and TAC. Outcomes are expressed as a difference between post‐ and pre‐treatment values. Center bars are medians; box tops and bottoms are interquartile ranges; whiskers are minimum and maximum values; crosses inside boxes are means. FMD, flow‐mediated dilatation; MDA, malondialdehyde; TAC, total antioxidant capacity

Baseline FMD values were not significantly different between the two groups (Table 1). The difference between post‐ and pre‐intervention FMD (ΔFMD) was significantly more in the melatonin than the placebo group (1.64% vs. −1.37% respectively, *p* = .029) with an effect size of 3.01% (95% CI: 0.32–5.70) (Figure 2). Also, when adjusted for pre‐treatment values and age, post‐treatment FMD was significantly associated with the intervention (estimated marginal means (EM) (95% CI): melatonin, 7.84% (6.69–8.98); placebo, 5.98% (4.84–7.12); *p* = .027). Adjustment for DM status led to the same results (Table 2).

**TABLE 2:** Univariate regression analysis of effect of melatonin supplementation on FMD

Outcomes	Estimated marginal means (95% CI)		Study groups *p* value^a^	Covariates *p* values
FMD (%)	Melatonin (n = 42)	Placebo (n = 43)		
	7.71 (6.55–8.86)	6.11 (4.96–7.26)	.056	Baseline FMD: 0.283
	7.84 (6.69–8.98)	5.98 (4.84–7.12)	.027	Baseline FMD: 0.135 Age: 0.052
	7.60 (6.40–8.79)	5.69 (4.45–6.93)	.031	Baseline FMD: 0.337 Age: 0.155 Diabetes status: 0.120

Abbreviation: FMD, flow‐mediated dilatation.

^a^ By one‐way analysis of covariance (ANCOVA).

### 3.4 Effect of melatonin supplementation on oxidative stress markers

Baseline values for the oxidative stress markers are shown in Table 1. In the unadjusted analysis, we assessed the differences between post‐ and pre‐intervention values between the groups. There was no statistically significant difference between the groups for the TAC (mean difference [MD] [95% CI]: 10.49[−81.05 to 102.03]). Nevertheless, the mean of the MDA was decreased in both groups, and the difference was significantly more in the melatonin group than the placebo group (MD [95% CI]: 1.94 [0.42–3.46]). Nonetheless, in the adjusted analysis for the baseline values, no association was found between the post‐treatment MDA and the intervention (EM [95% CI]: melatonin, 2.51 [1.76–3.26]; placebo, 3.19 [2.43–3.96]; *p* = .215).

### 3.5 Effect of melatonin supplementation on BP

Systolic and diastolic BP was measured three times during the study, but we had 20 missing at the first follow‐up, mainly because of the COVID‐19 pandemic. Thus, a generalized estimating equation analysis was used to include all available data. We did not find any association between systolic or diastolic BP with the treatment groups when adjusted for age and diuretic use. We included the diuretic use in the model, because this parameter was not equally distributed between study groups. Results for the BP analysis are shown in Table 3.

**TABLE 3:** Effect of melatonin supplementation on blood pressure

Outcome	Estimated marginal means (95% CI)		*p* value^a^
	Melatonin (n^b^ = 46)	Placebo (n = 46)	
Systolic blood pressure	121.7 (115.4–127.5)	116.0 (112.3–119.9)	Study group: 0.157 Age: 0.000 Diuretic use: 0.743
Diastolic blood pressure	76.1 (73.1–79.1)	72.4 (69.7–75.3)	Study group: 0.098 Age: 0.850 Diuretic use: 0.573

^a^ By Generalized Estimating Equations; age and diuretic use as covariates in the model.

^b^ Number of patients at baseline.

### 3.6 Subgroup analysis by DM status

**FIGURE 3:**
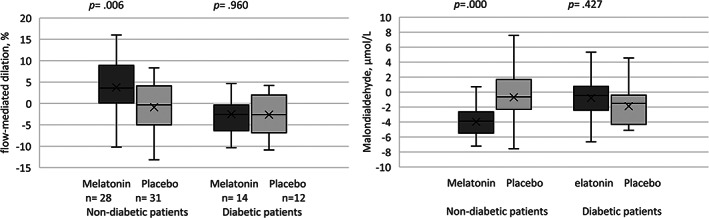
Subgroup analysis of effect of melatonin supplementation on FMD according to the diabetes status. Outcomes are expressed as a difference between post‐ and pre‐treatment values. Center bars are medians; box tops and bottoms are interquartile ranges; whiskers are minimum and maximum values; crosses inside boxes are means. FMD, flow‐mediated dilatation; MDA, malondialdehyde; TAC, total antioxidant capacity

We assessed the effect of the intervention on ΔFMD and ΔMDA in HF patients with or without DM. In nondiabetic patients, there was a significant difference between the melatonin and placebo groups in the mean of ΔFMD (MD (95%CI): 4.65% (1.41–7.88), *p* = .006) and ΔMDA (−3.28 μmol/L (−5.02 − [−1.55], *p* < .001). However, in diabetic patients no significant difference was seen according to the treatment groups (FMD: 0.10% [−4.27 to 4.06], *p* = .960; MDA: 1.10 μmol/L (3.91 − [−1.72]). Results of the subgroup analysis are shown in Figure 3.

## 4 DISCUSSION

It was demonstrated in this study that oral melatonin for 24 weeks had a beneficial effect on endothelial function in patients with HFrEF, but no effect on oxidative stress markers and systolic/diastolic BP, and its effect on endothelial function in DM patients was minimal. Up to our knowledge, this is the first study evaluating the effect of melatonin on endothelial function in HF.

The effect size of the intervention for FMD was about 2% after adjustment for covariates, which its clinical importance is really unknown. We did not measure the FMD at the first follow‐up; therefore, we cannot comment on the short‐term melatonin effect in these patients. Several studies have demonstrated that ED measured by FMD has a strong predictive value for adverse outcomes such as heart transplantation or cardiac death in patients with chronic HF.^15^, ^16^ Interestingly, it is shown that this risk is reduced with the improvement of FMD after optimum treatments.^17^ Thus, it could be inferred that melatonin can improve long‐term outcomes in HFrEF.

Melatonin has been shown to ameliorate ED in animal models of hypercholesterolemia and DM by regulating NO availability and inflammatory pathways;^18^, ^19^, ^20^ however, clinical trials results are inconsistent in this regard. In a randomized clinical trial, 1 month of 10 mg oral melatonin was found to reduce serum markers of ED and increase serum NO levels in patients with severe CAD.^21^ Also, 2 weeks of oral melatonin improved markers of ED in an animal model of smoke‐exposed and also in smoking human subjects who ingested daily 3 mg tablets of melatonin.^22^ In another study, while melatonin regulated some markers of ED in vitro and its high dose of 10 mg sustained‐release tablets, three times daily, had clinical benefit in early onset preeclampsia, but it did not affect endothelial function or oxidative stress markers in studied patients.^23^ Our results are also in contrast to a recent study, which demonstrated that 12 weeks of 25 mg oral melatonin did not improve endothelial function, measured by reactive hyperemia index, or its serum markers in patients after acute coronary syndrome.^24^

The reason for these discrepancies could be the various biomarkers used for oxidative stress and ED assessment, different target populations, and different doses and durations of melatonin administration, as it is stated that higher doses of melatonin might have a pro‐oxidant effect, at least in vitro.^25^

We did not find melatonin administration to affect the TAC, and its effect on the MDA was disappeared in the adjusted analysis. Melatonin reduces oxidative damage to biological molecules such as membrane polyunsaturated fatty acids,^5^ thus expected to reduce the MDA, which is the final product of their peroxidation. Our results regarding oxidative markers were relatively in agreement with a systematic review, which demonstrated that increased TAC by melatonin was more prominent in doses more than 20 mg and in people under 35 years old, but MDA was also decreased by lower doses and in older people.^26^

Nevertheless, it can be suggested that the beneficial effect of melatonin on the endothelium might be exerted via other protective pathways or might be reflected in other oxidative stress biomarkers not measured in this study.^27^

Melatonin is supposed to have a substantial antihypertensive effect because of central and peripheral mechanisms such as endothelium‐dependent vasodilation, antioxidant effects, and reducing sympathetic tone.^8^ This BP‐lowering effect was demonstrated in a systematic review to be more in patients with psychotic disorders than in patients with metabolic syndrome.^28^ The effect of melatonin on BP in patients with HF is not clear. In HFrEF, BP does not have a linear correlation with the patient outcomes, and in contrast with ischemic heart disease, lower BP could be associated with worse outcomes.^29^ Moreover, guideline‐recommended treatments in HFrEF all have antihypertensive effects; hence management of treatment protocols to prevent hypotension is a dilemma in these patients.^29^

We found that melatonin did not significantly affect BP in HFrEF, and adjunct administration of it with essential HF drugs might not interfere with BP regulation in these patients. Although, because of the circadian effects of melatonin on BP, it is recommended to investigate its effect via a 24‐h monitoring system.

It should be noted that our insignificant results in oxidative markers and BP might be due to low‐sample size, diversity of clinical and demographic characteristics of our participants, or subtle imbalances in baseline values of the variables. However, our patients seem to be an acceptable representative sample of HFrEF.

A subgroup analysis in nondiabetic patients showed better results for the FMD and MDA relative to the mixed population. Conversely, no significant difference was seen between study groups in diabetic patients. This may suggest that our intervention did not benefit the diabetic group. Although the low‐sample size of this study for diabetic HF patients prevents us from a definitive conclusion and it is recommended to test this hypothesis in larger studies, as up to our knowledge, no clinical study in this regard has been published.

Indeed, our study's prominent shortcoming is the low‐sample size relative to the natural heterogeneity of HFrEF patients. However, this is a phase II study, and our primary outcome has been met with this sample size. Another limitation of this study is the missing data of the first follow‐up, which was unavoidable because of the unusual situation created by the COVID‐19 pandemic, and we tried to compensate for it by the statistical methods. Also, as it is noted in Table 1, there is some imbalances in current HF drugs used in two study groups, especially for diuretic use; however, the main drugs affecting FMD are balanced between the groups and the analysis for BP assessment were adjusted for diuretic use to get accurate results.

On the other hand, this is the first study of melatonin's effect on the endothelial function in HF, and its design as a double‐blinded randomized clinical trial and the long duration of the follow‐up relative to the other related studies are its strengths.

In conclusion, melatonin might be beneficial for the endothelial function in patients with HFrEF, and as a consequence, it could improve their outcome. Nevertheless, according to our results, this benefit in diabetic patients with HFrEF is debated and should be examined in a properly designed study.

## CONFLICT OF INTEREST

The authors declare that they have no conflict of interest.

## Data Availability

The data that support the findings of this study are available from the corresponding author upon reasonable request.

## References

[1] Zuchi C, Tritto I, Carluccio E, Mattei C, Cattadori G, Ambrosio G. Role of endothelial dysfunction in heart failure. Heart Fail Rev. 2020;25(1):21‐30. 31686283 10.1007/s10741-019-09881-3

[2] Canetti M, Akhter MW, Lerman A, et al. Evaluation of myocardial blood flow reserve in patients with chronic congestive heart failure due to idiopathic dilated cardiomyopathy. Am J Card. 2003;92(10):1246‐1249. 14609613 10.1016/j.amjcard.2003.08.002

[3] Alem MM. Endothelial dysfunction in chronic heart failure: assessment, findings, significance, and potential therapeutic targets. Int J Mol Sci. 2019;20(13):3198. 10.3390/ijms20133198 PMC6651535 31261886

[4] Areas GPT, Mazzuco A, Caruso FR, et al. Flow‐mediated dilation and heart failure: a review with implications to physical rehabilitation. Heart Fail Rev. 2019;24(1):69‐80. 29995216 10.1007/s10741-018-9719-7

[5] Galano A, Reiter RJ. Melatonin and its metabolites vs oxidative stress: from individual actions to collective protection. J Pineal Res. 2018;65(1):e12514. 29888508 10.1111/jpi.12514

[6] Mauriz JL, Collado PS, Veneroso C, Reiter RJ, González‐Gallego J. A review of the molecular aspects of melatonin's anti‐inflammatory actions: recent insights and new perspectives. J Pineal Res. 2013;54(1):1‐14. 22725668 10.1111/j.1600-079X.2012.01014.x

[7] Jafari‐Vayghan H, Saleh‐Ghadimi S, Maleki V, Moludi J, Alizadeh M. The effects of melatonin on neurohormonal regulation in cardiac cachexia: a mechanistic review. J Cell Biochem. 2019;120(10):16340‐16351. 31168891 10.1002/jcb.29151

[8] Pechanova O, Paulis L, Simko F. Peripheral and central effects of melatonin on blood pressure regulation. Int J Mol Sci. 2014;15(10):17920‐17937. 25299692 10.3390/ijms151017920 PMC4227197

[9] Rodella LF, Favero G, Foglio E, et al. Vascular endothelial cells and dysfunctions: role of melatonin. Front Biosci. 2013;5:119‐129. 10.2741/e601 23276975

[10] Konukoglu D, Uzun H. Endothelial dysfunction and hypertension. Adv Exp med Biol. 2017;956:511‐540. 28035582 10.1007/5584_2016_90

[11] Sadeghi M, Khosrawi S, Heshmat‐Ghahdarijani K, et al. Effect of melatonin on heart failure: design for a double‐blinded randomized clinical trial. ESC Heart Fail. 2020;7(5):3142‐3150. 32618134 10.1002/ehf2.12829 PMC7524054

[12] Thijssen DH, Black MA, Pyke KE, et al. Assessment of flow‐mediated dilation in humans: a methodological and physiological guideline. Am J Physiol Heart Circ Physiol. 2011;300(1):H2‐H12. 20952670 10.1152/ajpheart.00471.2010 PMC3023245

[13] Oikonomou E, Vogiatzi G, Karlis D, et al. Effects of omega‐3 polyunsaturated fatty acids on fibrosis, endothelial function and myocardial performance, in ischemic heart failure patients. Clin Nutr. 2019;38(3):1188‐1197. 29752009 10.1016/j.clnu.2018.04.017

[14] Kadam P, Bhalerao S. Sample size calculation. Int J Ayurveda Res. 2010;1(1):55‐57. 20532100 10.4103/0974-7788.59946 PMC2876926

[15] Meyer B, Mörtl D, Strecker K, et al. Flow‐mediated vasodilation predicts outcome in patients with chronic heart failure: comparison with B‐type natriuretic peptide. J Am Coll Cardiol. 2005;46(6):1011‐1018. 16168284 10.1016/j.jacc.2005.04.060

[16] Tarro Genta F, Eleuteri E, Temporelli PL, et al. Flow‐mediated dilation normalization predicts outcome in chronic heart failure patients. J Card Fail. 2013;19(4):260‐267. 23582092 10.1016/j.cardfail.2013.01.014

[17] Takishima I, Nakamura T, Hirano M, et al. Predictive value of serial assessment of endothelial function in chronic heart failure. Int J Cardiol. 2012;158(3):417‐422. 21371765 10.1016/j.ijcard.2011.01.059

[18] Sezgin D, Aslan G, Sahin K, et al. The effects of melatonin against atherosclerosis‐induced endothelial dysfunction and inflammation in hypercholesterolemic rats. Arch Physiol Biochem. 2020;1‐8. 10.1080/13813455.2020.1838550. 33156709

[19] Salmanoglu DS, Gurpinar T, Vural K, Ekerbicer N, Darıverenli E, Var A. Melatonin and L‐carnitin improves endothelial disfunction and oxidative stress in type 2 diabetic rats. Redox Biol. 2016;8:199‐204. 26803481 10.1016/j.redox.2015.11.007 PMC4731948

[20] Hu ZP, Fang XL, Fang N, et al. Melatonin ameliorates vascular endothelial dysfunction, inflammation, and atherosclerosis by suppressing the TLR4/NF‐κB system in high‐fat‐fed rabbits. J Pineal Res. 2013;55(4):388‐398. 24006943 10.1111/jpi.12085

[21] Javanmard SH, Heshmat‐Ghahdarijani K, Mirmohammad‐Sadeghi M, Sonbolestan SA, Ziayi A. The effect of melatonin on endothelial dysfunction in patient undergoing coronary artery bypass grafting surgery. Adv Biomed Res. 2016;5:174. 28028514 10.4103/2277-9175.194801 PMC5156974

[22] Wang Z, Ni L, Wang J, et al. The protective effect of melatonin on smoke‐induced vascular injury in rats and humans: a randomized controlled trial. J Pineal Res. 2016;60(2):217‐227. 26681403 10.1111/jpi.12305

[23] Hobson SR, Gurusinghe S, Lim R, et al. Melatonin improves endothelial function in vitro and prolongs pregnancy in women with early‐onset preeclampsia. J Pineal Res. 2018;65(3):e12508. 29766570 10.1111/jpi.12508

[24] Zahid JA, Isbrand A, Kleif J, et al. The effect of melatonin on endothelial dysfunction in patients after acute coronary syndrome: the MEFACS randomized clinical trial. J Pineal Res. 2019;67(3):e12600. 31355944 10.1111/jpi.12600

[25] Zhang HM, Zhang Y. Melatonin: a well‐documented antioxidant with conditional pro‐oxidant actions. J Pineal Res. 2014;57(2):131‐146. 25060102 10.1111/jpi.12162

[26] Ghorbaninejad P, Sheikhhossein F, Djafari F, et al. Effects of melatonin supplementation on oxidative stress: a systematic review and meta‐analysis of randomized controlled trials. Horm Mol Biol Clin Investig. 2020;41(4):20200030. 10.1515/hmbci-2020-0030 33185572

[27] Frijhoff J, Winyard PG, Zarkovic N, et al. Clinical relevance of biomarkers of oxidative stress. Antioxid Redox Signal. 2015;23(14):1144‐1170. 26415143 10.1089/ars.2015.6317 PMC4657513

[28] Hadi A, Ghaedi E, Moradi S, Pourmasoumi M, Ghavami A, Kafeshani M. Effects of melatonin supplementation on blood pressure: a systematic review and meta‐analysis of randomized controlled trials. Horm Metab Res. 2019;51(3):157‐164. 30861561 10.1055/a-0841-6638

[29] Pinho‐Gomes AC, Rahimi K. Management of blood pressure in heart failure. Heart. 2019;105(8):589‐595. 30674544 10.1136/heartjnl-2018-314438

